# Directory for Dental Societies and Colleges

**Published:** 1899-01

**Authors:** 


### Directory for Dental Societies and Colleges.

The Dental Register has for several years published a list of dental societies of the United States, embracing the State Societies and the more important local Societies.

The request and expectation at the outstart was that the officers of these societies should send to the editor all changes and data for maintaining this directory correct and complete. This expectation has not been fully answered. Occasionally

THE REGISTER has sent out a request to all societies for their co-operation in this matter. These requests have been generally regarded, for the time being at least, but in many instances proper care is not exercised in this matter, so that frequently the time and place of meeting and the officers as given in this directory were incorrect.

As a matter of reference such a directory is desirable and should be accurate, and we earnestly request that all persons connected with societies in an official capacity will see to it that we have the proper data for their meetings.

In regard to the directory for colleges changes do not so frequently occur, still we shall be pleased to have the co-operation of all faculties of colleges to maintain this directory complete. Both of these are matters of great convenience to the profession, at least expressions of this sort so frequently given as to warrant the publication, so please furnish us with all changes and data in respect to these organizations.

